# The Anatomical Remembrancer, or Complete Pocket Anatomist

**Published:** 1845-12

**Authors:** 


*The Anatomical Remembrancer, or Complete Pocket Anatomist,* is the title of a re-publication from the second London edition, just received from the publishers, Samuel S. & William Wood, New York.

This little manual, so condensed as to require but an hour or two for its perusal, contains a concise and clear description of all the principal anatomical structures; and is admirably adapted to the purpose for which it was intended—to recall to mind anatomical knowledge already acquired. For such a purpose we cheerfully recommend it to students, and to practitioners without leisure, wishing, occasionally to refresh the memory with some of the most prominent and useful anatomical facts. H.

